# Supplementary material for: Are carbon emissions trading and green financial instruments synergistic? -Comprehensive quantitative research based on content analysis
Source: PLoS One. 2024 Mar 7;19(3):e0298601. doi: 10.1371/journal.pone.0298601 (PMC10919601; doi:10.1371/journal.pone.0298601)
Supplement: S1 Data — (ZIP) [file pone.0298601.s001.zip › Data/地级市Z_F工作报告.zip!/╕╩╦α/╝╬╙°╣╪╩╨/└┤╫╘╣1⁄2╓┌║┼í╛┬φ┐╦╩2╛▌═°í┐_2015.docx]

各位代表：

　　现在，我代表市人民政府，向大会作工作报告，请予审议，并请政协委员和列席人员提出意见。

　　2014年工作回顾

　　过去的一年，在省委、省政府和市委的坚强领导下，我们深入贯彻落实党的十八大和十八届三中、四中全会精神，坚持稳中求进、改革创新的工作总基调，积极应对复杂严峻的外部环境和持续加大的经济下行压力，全力做好稳增长、调结构、促改革、惠民生各项工作，经济社会实现了平稳健康发展。预计完成生产总值260亿元，增长12.5%；规模以上工业增加值148亿元，增长14.5%。城镇居民人均可支配收入26894元，增长10.7%；农民人均纯收入13809元，增长11.8%；全社会固定资产投资126亿元，增长30%；社会消费品零售总额43.3亿元，增长14%；公共财政预算收入15.8亿元，增长3.2%；地区性财政收入50.9亿元，增长26.3%；居民消费价格总水平涨幅控制在2.6%，较好地完成了市九届人大三次会议确定的目标任务。

　　（一）大力调整产业结构，发展质量不断提升

　　坚持把项目建设作为调结构、转方式的重要抓手，优化 “3341”项目库，全年实施重点项目78项。

　　工业转型取得突破。全力支持酒钢集团公司深化内部改革，循环经济和产业结构调整项目顺利推进，1000万吨煤炭分质利用项目开工建设，铝产业产能达到135万吨，在市场低迷和主要产品价格持续下跌的情况下，实现了扭亏为盈。工业园区配套设施进一步完善，服务功能进一步增强，循环经济加快发展，被确定为国家低碳试点园区；广银铝业铝模板、中威斯冶金新材料等项目建成；嘉西光伏产业园被列为全省7个百万千瓦级光伏发电基地之一，中利腾晖、汉吉国能光伏电站和110千伏汇集升压站等项目稳步推进。

　　第三产业增速明显。坚持以文化带旅游，以旅游促商贸，着力促进文化旅游商贸融合发展。世界文化遗产保护与展示工程基本完成，方特欢乐世界主体建成，文化产业实现增加值3.8亿元，增长28%。加大旅游宣传促销力度，完善基础设施，提升服务质量，成功创建全国旅游标准化示范城市，全社会旅游人数和旅游收入均增长26%。加快重点区域商贸中心建设，观礼古镇一期商业步行街和天诚国际购物中心主体封顶，富力城市综合体顺利推进，恒基美居家居生活广场即将开业。金融保险、电子商务、健康服务等现代服务业快速发展，第三产业比重上升了6.2个百分点，较好地实现了发展提速、比重提高和水平提升的预期目标。

　　现代农业加快发展。围绕农业增效、农民增收，实施农业项目23项，完成固定资产投资1.6亿元。正大现代农业合作示范项目一期投入使用；新城镇、峪泉镇4万亩高效节水项目扎实推进；高标准农田综合开发、野麻湾农业高新示范园等项目进展顺利。加大优势产业培育力度，新建日光温室162座、养殖小区16个，各类农村专业合作社达到190家，优质蔬菜、果品、食用菌等特色产业快速发展。农村土地流转有序进行，流转面积近1.2万亩，占耕地总面积的17%。

　　（二）统筹推进城乡建设，人居环境持续改善

　　坚持生态立市战略，以推进新型城镇化为抓手，进一步完善城乡基础设施，强化生态环境保护和建设，居民生活品质不断提高。

　　城乡规划建设扎实推进。坚持一张蓝图绘到底，依据总体规划，编制实施了南市区控制性详规和商业、消防、公共服务、绿地等专项规划；加快推进南市区建设，道路、供排水、停车场等设施不断完善；火车南站及站前广场如期交付使用，保障了兰新高铁顺利开通运行。旧城区综合整治有序推进，对部分小区实施了供热、污水管网、绿化、亮化改造。出台了棚户区改造总体方案，惠民街区改造顺利启动。城镇化建设步伐加快，农村危旧房屋改造、村容环境整治、垃圾收集清运和污水处理等项目稳步推进，道路、路灯、环卫等基础设施运行维护得到加强。

　　城市管理水平不断提高。以创建全国文明城市为目标，实施市民素质提升、交通秩序优化和市容市貌整治专项行动，城市品位不断提升。积极巩固国家卫生城市创建成果，顺利通过第二次复查确认。深入开展“物业管理服务年”活动，小区物业规范化管理合格率达到92%。新购置公交车24辆，线路达到12条，实现了城市社区、车站、商业聚集区、重点景区的全覆盖。扎实推进“智慧城市”建设，数字嘉峪关地理空间框架建设项目通过验收，成功入围全省首批数字城市建设推广市。

　　生态环境质量持续改善。按照“一年打基础、两年见成效、三年促完善”的总体目标，实施了酒钢宏晟电热脱硫脱硝等13项污染减排项目，加大对工业废气、扬尘、煤炭和汽车尾气等重点污染源的整治力度，加强对嘉北区域煤场、料场监管，环境空气质量逐步改善。严格抓好节能降耗，单位生产总值能耗、主要污染物排放等约束性指标完成预定目标。积极创建国家园林城市，新建绿地154公顷，建成区绿地率、绿化覆盖率分别达到37.6%和38.8%，人均公共绿地面积36.3平方米，比上年增加2.4平方米。讨赖河南岸输水工程建成使用，饮用水源井迁建项目进展顺利。

　　（三）不断深化改革开放，发展活力有效增强

　　坚持把改革开放作为激发活力的关键，各项改革任务扎实推进，扩大开放取得新的进展。

　　全面深化改革步伐加快。稳步推进政府机构改革，根据省委、省政府统一部署，整合组建了市场监管局、综合执法局、社会治理局和卫生计生委。加快转变政府职能，承接省政府下放行政审批事项53项，取消27项，备案管理5项，保留的审批事项全部进驻政务服务中心。全面落实促进非公经济发展的各项政策措施，工商注册登记制度改革顺利完成，新增市场主体4008户；“营改增”试点受益企业达到795户，实现直接减税2191万元；非公经济完成增加值50亿元，增长23%。深化投融资体制改革，成功发行10亿元企业债券，农村合作银行改制为农村商业银行，银行业金融机构各项贷款余额388.5亿元，增长10%，中小微企业贷款余额增长41%。

　　扩大对外开放成效明显。抢抓“丝绸之路经济带”历史机遇，区域循环经济及能源综合利用合作得到加强，对外开放不断深化。嘉峪关国际港务区前期工作进展顺利，机场航空口岸开放列入全省旅游交通运输发展总体布局，货邮吞吐量达到1130吨，增长20%，开通航线达到16条。第20届兰洽会我市签约总额170.5亿元，全年招商引资到位资金增长31.8%。

　　（四）切实强化民生保障，公共服务更加完善

　　坚持民生优先，加大民生投入，全年民生支出15.8亿元，占公共财政预算支出的71.6%。

　　民生和社会事业加快发展。全年新增城镇就业7500人，城镇登记失业率控制在2.7%以内。城乡居民社会养老保险参保率达到97.1%，新农合年人均补助标准高出全省平均水平50元；下调了城镇职工大病医疗保险起付标准，提高了年度最高支付限额。新建各类保障性住房660套。义务教育均衡发展工作通过国家评估，职业教育快速发展，高中阶段毛入学率达到95%以上。基层医疗条件明显改善，公共卫生应急和传染病防控工作得到加强。雄关大舞台、乡村舞台等群众性文化活动深入开展。成功举办了第10次铁人三项赛暨世界杯赛。积极支持驻嘉部队和国防后备力量建设，双拥共建取得新成效。

　　惠民实事基本完成。残疾人免费乘坐公交车政策全面落实；为1584名重度残疾人发放护理补贴125万元，为200户重度残疾人家庭进行了无障碍设施改造。居家养老服务中心投入使用，注册老人达到1.7万人，日均服务超过1300人次；上门医疗服务实现城乡全覆盖。3所农村中小学教学楼加固改造项目完工，明珠学校初中部建设项目完成招标。6个城乡标准化社区服务中心、3个农超对接超市项目进展顺利，10个体育健身场地全部建成。兰新、昌明小区环境综合改造顺利完成。完成5万户广电网络双向改造任务。新建改造的10座公共卫生间建成投用9座，在建1座。

　　社会治理方式不断改进。平安嘉峪关建设稳步推进，社会信用体系建设全面启动，保持对违法犯罪的严打高压态势，及时处理突出信访问题和群体性事件，律师进社区工作深入开展，群众安全感进一步增强，在全省公共安全感地区排名中位列第一，连续第4年被省委、省政府评为“全省综治（平安建设）优秀地区”。不断加大产品质量、食品药品、物价等监管力度，有力维护了市场秩序。全面落实安全生产责任体系，各类安全生产事故起数下降19.4%。

　　与此同时，粮食、供销、邮政、通信、人防、统计、档案、市志、地震、气象、老龄、慈善、民族宗教、外事侨务、人口与计划生育、妇女儿童等工作也都取得新的进展。

　　（五）着力改进工作作风，自身建设得到加强

　　围绕建设为民务实清廉政府，深入开展党的群众路线教育实践活动，作风建设和廉政建设不断加强。

　　作风建设取得实效。全面实施“找准钉点、研究钉准、保证钉牢”的为民务实“三步钉钉法”，解决了一批群众关心、关注的热点难点问题。深入开展窗口单位优质服务行动，为民服务更加规范、高效。严格执行中央、省、市作风建设各项规定，撤销议事协调机构209个，在政府系统实现了无纸化公文传递，严控一般性支出，“三公”经费降低8.1%。

　　依法行政工作深入推进。坚持常务会议学法制度，修订完善了《政府工作规则》，出台了行政程序规定，成立了法律专家咨询委员会，严格按照法律赋予的权力行使职权、履行职责。自觉接受人大法律监督和政协民主监督，认真办理答复人大建议、政协提案，办复率100%。深入推进政务公开和政府信息公开，主动接受舆论和群众监督，全年主动公开信息3.6万条。

　　党风廉政建设不断加强。认真落实党风廉政建设主体责任，加强重点领域专项检查和行政效能电子监察，规范公共资源交易行为，深入开展廉政风险防控，对政府投资项目全部实行代建制管理。全市所有预算单位公开了部门预决算及“三公经费”支出情况。

　　各位代表，过去的一年，在经济下行压力加大、外部环境错综复杂、困难矛盾不断增多的情况下，取得这样的成绩来之不易。这是省委、省政府亲切关怀、市委正确领导的结果，是人大、政协依法有效监督的结果，是嘉峪关军分区、酒钢集团公司、中核四〇四有限公司及驻嘉各单位、社会各界大力支持的结果，是全市上下同心协力、拼搏进取的结果。在此，我代表市人民政府，向全市人民群众，向各民主党派、工商联、人民团体、离退休老同志，向驻嘉部队、武警官兵和各驻嘉单位致以崇高的敬意！向所有关心和支持嘉峪关发展的社会各界人士表示衷心的感谢!

　　各位代表，回顾过去一年的工作，在肯定成绩的同时，也要清醒地看到，全市经济社会发展中还存在不少困难和问题。主要表现在：受宏观经济形势影响，主要经济指标增速回落，财政收支矛盾突出；经济结构调整任务依然艰巨，部分企业生产经营困难，发展质量和效益有待提升；资源、环境和要素制约进一步趋紧，大气污染、节能减排等环境保护治理工作还需要加大力度；政府职能转变还不够到位，工作作风和办事效率仍需改进，依法履职的能力和水平有待提高。这些问题，我们要高度重视，努力用改革的思路和创新的办法切实加以解决。

　　2015年主要任务

　　2015年是全面深化改革的关键之年，是全面推进依法治国的开局之年，也是全面落实“十二五”规划的收官之年。今年政府工作的总体要求是：深入贯彻党的十八大和十八届三中、四中全会精神，全面落实中央、省、市经济工作会议和省十二届人大三次会议提出的各项要求，主动适应经济发展新常态，坚持稳中求进工作总基调，以提高发展质量和效益为中心，把转方式、调结构放到更加重要的位置，狠抓改革攻坚，强化创新驱动，推进转型升级，保持经济社会持续健康发展、社会和谐稳定，努力加快全面建成小康社会进程。

　　今年经济社会发展主要预期目标是：地区生产总值增长9%左右，规模以上工业增加值增长9%左右，全社会固定资产投资增长21%左右，社会消费品零售总额增长13%左右，公共财政收入预算增长10%左右，城镇居民人均可支配收入增长9%左右，农民人均纯收入增长11%左右，居民消费价格指数涨幅控制在3%以内。

　　上述目标是在主动适应经济发展新常态，统筹考虑稳增长、抓改革、促调整、惠民生、防风险等各方面因素的基础上提出的。核心目的就是要把经济增长保持在更加合理的区间内，更好地推进结构调整、保障和改善民生、促进社会和谐稳定，为全市经济社会持续健康发展积蓄力量，确保各项预期目标顺利完成，并在此基础上争取发展的更好一些，更快一些。

　　围绕上述目标，重点做好五个方面的工作：

　　（一）加快产业转型升级，努力保持经济稳定增长

　　深入实施“3341”项目工程，加快经济结构调整和产业转型升级，增强综合经济实力。

　　深入实施工业强市战略。充分发挥冶金产业优势和酒钢集团公司带动作用，集聚整合资源，引进战略投资，着力构建“煤炭—发电—冶金—精深加工—装备制造”千亿级循环经济产业链。全力支持酒钢深化改革和多元化发展，加快循环经济和结构调整、1000万吨煤炭分质利用等重点项目建设，形成钢铁、电解铝、电力能源与煤化工产业相互促进、相互支撑、循环利用的低碳高效耦合发展格局。扎实推进嘉峪关国家低碳工业园区试点，进一步加大基础设施配套和环境整治力度，加快中威斯冶金新材料二期、索通34万吨预焙阳极、广银30万吨铝模板等项目建设。加大嘉西光伏产业园在建发电项目建设力度，做好黄草营330千伏变电站及送出工程建设工作，加快推进百万千瓦级光伏发电基地建设。继续做好过剩产能化解工作，完成省政府节能减排、淘汰落后产能各项任务。

　　大力发展文化旅游产业。依托华夏文明传承创新区平台，加快推进丝绸之路非物质文化遗产古镇、长城边关第一镇、南湖文化生态园、草湖国家湿地公园、戈壁大峡谷地质公园等重点项目建设，实现文化产业集群发展。强化协调服务，完善设施配套，加大宣传推介，确保方特欢乐世界在“五一”顺利开业运营。深入开展“旅游服务提升年”行动，完善旅游基础设施、强化从业人员培训，鼓励发展乡村旅游、星级农家乐、家庭旅馆等富民项目，优化旅游环境，增强服务水平和综合承载能力。大力发展商贸、餐饮、娱乐、休闲等产业，抓好观礼古镇、雄关宾馆和五一商场改造等项目建设，力争把我市建成全省文化旅游休闲消费型城市。

　　加快发展现代高效农业。以提高农业综合效益和市场化程度为核心，鼓励发展高效特色优势农业。深入推进正大现代农业合作示范等项目建设，发挥龙头引领作用，加快产业化种植基地和标准化养殖小区建设。为农村集体资产确权登记发证，引导农村居民依法自愿有偿流转土地，加快培育专业大户、家庭农场、农民合作社等新型经营主体。完善农产品销售流通网络，扶持发展农村电子商务，推动现代高效农业发展。继续深入开展“双联”行动，切实为群众解决实际问题。

　　（二）加大改革攻坚力度，不断增强发展内生动力

　　以政府自身改革为引领，进一步破除体制机制障碍，狠抓各领域改革，力求取得新的进展。

　　积极推进政府机构改革。深化行政审批制度改革，继续做好行政审批事项接放管。全面实施政府行政权力清单、责任清单、市级财政专项资金管理清单和政务服务网“三张清单一张网”建设，加强事中事后监管，着力建设有限、有为、有效的服务型政府。扩大政府购买服务范围，提高政府公共服务供给效率和质量。理顺市场监管局、综合执法局、社会治理局、卫生计生委等部门工作职能，全面履行职责，提高工作效能。规范公共资源交易行为，发挥市场在资源配置中的决定性作用，着力营造规范、透明、高效的市场环境。

　　统筹推进其他领域改革。全面落实工商登记制度改革等措施，营造公平竞争的市场环境，促进非公经济加快发展。继续落实“营改增”、结构性减税等税收优惠政策，进一步减轻中小微企业税负。搭建好政银企对接平台，实施差别化金融支持政策，更大力度扶持小微企业发展。全面推进社会、财税、农村、科技、水利等各项改革，着力激发经济社会发展活力。

　　构建开放型经济新格局。积极融入国家“一带一路”发展战略，支持企业统筹利用国际、国内“两个市场、两种资源”，加强对外合作交流。办好第四届“中国嘉峪关国际短片电影展”和第五届“敦煌行• 丝绸之路国际旅游节”开幕式及相关活动，提升城市影响力。强化区域合作，继续推进嘉酒经济一体化进程，推动周边区域循环经济及新能源产业合作，实现协同发展。加快嘉峪关国际港务区建设，推进保税物流中心申报进程，申请嘉峪关航空口岸开放。围绕结构调整重点，抓好招商引资，强化跟踪服务，不断增强竞争力。

　　（三）统筹推进城乡建设，着力提升新型城镇化水平

　　坚持以人为本、生态优先，引领城市空间扩展，完善功能布局，进一步提升城市品位。

　　着力完善城乡基础设施。坚持规划先行，进一步完善城乡规划体系，彰显历史文化特色，形成人口、产业、资源和环境相协调的格局。稳步推进南市区基础设施建设，新建酒钢热电厂至南市区一级供热管网，开工建设金港路跨清嘉高速桥梁工程。加快旧城区供热、排水、燃气等基础设施和棚户区改造步伐。全面落实统筹城乡发展的各项政策，促进城乡公共服务均等化。抓好峪泉镇国家级新型城镇化试点，推进嘉峪关村、石桥村美丽乡村省级示范点建设。实施好综合客运枢纽、长城旅游公路、双拥路口公铁立交、嘉峪关至黑鹰山战备公路二期等交通项目，加快嘉峪关至天峻铁路（公路）等项目申报工作，提高区域交通保障能力。

　　加强城市精细化管理。深入推进“智慧城市”建设，加快宽带网络升级改造，整合城市信息化平台资源，提高城市管理的信息化水平。加强城市综合执法，深入开展城市交通、市容环境等专项行动，重点整治占道经营、违章停车、乱贴乱画、乱倒垃圾等现象，净化城市环境。继续规范物业管理，不断提升物业服务质量和水平。巩固创建国家文明城市成果，引导广大市民积极参与城市管理，推动全社会形成遵规、礼让、守序的良好风尚。

　　持续优化人居环境。严格执行新环保法，深入开展环境保护和大气污染治理行动，健全完善源头严控、过程严管、后果严惩的机制，倒逼产业转型，优化城乡环境。全面推进工业“三废”、煤炭、扬尘、机动车尾气等重点污染源治理，加大嘉北区域料场、煤场、施工场地和道路扬尘污染综合整治，禁止在城市中心区域和文物保护区设立煤炭经营场所，加快黄标车淘汰进度，加强燃煤小锅炉管控，重拳打击违法排污，确保实现大气污染防治“两年见成效”的目标。继续推进城乡绿化工程建设，提升道路、居民区绿化和管护水平，积极争创国家园林城市。实行最严格的水资源管理制度，加快实施高效节水灌溉工程，完成饮用水源井迁建等项目建设，建立健全安全高效的用水机制。

　　（四）持续抓好民生改善，切实增进民生福祉

　　牢固树立群众想的就是我们要干的、群众的难点和热点就是我们工作重点的理念，坚持民生优先，不断提升人民群众幸福指数。

　　稳步提高保障水平。统筹解决好高校毕业生、农村转移劳动力、退伍士兵等重点人群就业问题，城镇新增就业7500人，城镇登记失业率控制在4%以内。完善城乡医疗救助制度，提高临时救助水平，推进机关事业单位养老保险制度改革。完善居家养老服务体系和机制，基本实现居家养老服务城乡全覆盖。为全市75岁以上老人发放高龄补贴。

　　繁荣发展社会事业。深化教育领域综合改革，推进义务教育薄弱学校改造，开工建设明珠学校初中部、市四中综合楼，完成市酒钢三中搬迁招生工作。加快发展现代职业教育，支持甘肃钢铁职业技术学院改革发展。深化医药卫生体制改革，不断优化卫生资源配置，推进医师多点执业。继续办好雄关大舞台、农村文化大院等群众性文化活动。争创双拥模范城“六连冠”。

　　努力维护社会稳定。深入推进平安嘉峪关建设，加快社会信用体系建设步伐，规范市场经济秩序。继续推进标准化社区服务中心建设，以网格化管理、社会化服务为方向，促进社区管理规范化。扎实推进“六五普法”，依法严厉打击各类违法犯罪活动，积极开展矛盾纠纷排查和化解工作，预防和减少各种不安全、不稳定因素。加强重点领域、重点行业安全隐患排查整治，全面开展突发公共事件、防灾减灾等应急演练，确保社会和谐稳定。高度重视食品药品监管工作，完善覆盖全过程和产业链各环节的食品药品监管制度，确保人民群众“舌尖上的安全”。

　　全力办好惠民实事。1.完成惠民街区棚户区改造工作。2.为全市所有公办学校、幼儿园配置净化加热饮水设备。3.开工建设市中医院综合楼，完成南市区医院门诊急诊楼主体工程。4.加强城乡公共安全应急能力建设，强化公共卫生应急物资储备，配备水质监测设备；完善农产品质量安全检验检测体系。5.再实施200户重度残疾人家庭无障碍设施改造工程，为重度残疾儿童提供康复治疗服务，为重度残疾人发放护理补贴。6.对迎宾二、四小区进行环境综合整治。7.实施文化路改造提升工程。8.新建改造6个城市标准化社区服务中心，配套建设农超对接平价店等便民服务设施。9.建设10个城乡社区体育健身中心；新增3台智能图书借阅机。

　　（五）深入推进自身建设，努力建设为民务实清廉政府

　　巩固和发扬党的群众路线教育实践活动成果，以转作风、抓落实的实际成效取信于民。

　　持之以恒转变作风。认真贯彻执行中央、省、市作风建设各项规定，进一步加强调查研究，密切联系群众，持续改进文风会风。牢固树立“过紧日子”的思想，严格控制“三公”经费，节约一切财力为群众办好事、办实事。坚决纠正不作为、乱作为、慢作为，以抓铁有痕、踏石留印的精神狠抓工作落实，着力提升政府工作的执行力。

　　始终坚持依法行政。全面落实依法治国基本方略，自觉接受人大法律监督、政协民主监督和社会各界监督，认真办理人大建议和政协提案。坚持依法、科学、民主决策，把公众参与、专家论证、风险评估、合法性审查、集体讨论决定作为重大行政决策必经程序，严格按照法定权限和程序行使权力、履行职责。深入推进政务公开、办事公开，让政府权力在阳光下运行。

　　不断强化党风廉政建设。全面落实党风廉政建设主体责任，严格执行廉政建设各项规定，加强对重点领域、重点工程和关键环节的监管，切实规范权力行使，着力从源头上预防和治理腐败。坚持严管理、严要求，教育引导政府系统广大干部讲政治、顾大局、守纪律，始终保持昂扬斗志、浩然正气，努力建设为民务实清廉政府。

　　各位代表！

　　编制“十三五”经济社会发展规划是今年的一项重要工作。我们将问政于民、问计于民、问需于民，科学论证规划的总体思路、主要目标和重点任务，切实做到符合实际、尽力而为、量力而行，使规划成为指导未来五年全市经济社会科学发展的纲领性文件。

　　各位代表！

　　面对发展新形势、人民新期待，我们深感责任重大、使命光荣。让我们在省委、省政府和市委的坚强领导下，团结依靠全市人民，解放思想、改革创新，同心同德、奋力拼搏，为率先全面建成小康社会而努力奋斗！
